# Agmatine for Pain Management in Dogs With Coxofemoral Joint Osteoarthritis: A Pilot Study

**DOI:** 10.3389/fvets.2018.00311

**Published:** 2018-12-12

**Authors:** Takashi Taguchi, Ronald Koh, Catherine Takawira, Nathalie Rademacher, Gad M. Gilad, Randy D. Aronson, Mandi J. Lopez

**Affiliations:** ^1^Laboratory for Equine and Comparative Orthopedic Research, Department of Veterinary Clinical Sciences, School of Veterinary Medicine, Louisiana State University, Baton Rouge, LA, United States; ^2^Department of Veterinary Clinical Sciences, School of Veterinary Medicine, Louisiana State University, Baton Rouge, LA, United States; ^3^Gilad & Gilad LLC, Henderson, NV, United States; ^4^P.A.W.S. (Partners in Animal Wellness Services) Veterinary Center, Tucson, AZ, United States

**Keywords:** Agmatine, coxofemoral joint, hip dysplasia, osteoarthritis, intervertebral disc, gait, ground reaction force, canine

## Abstract

**Background:** Pain from coxofemoral joint (CFJ) osteoarthritis (OA) characteristic of canine hip dysplasia (CHD) afflicts many dogs. Intervertebral disc (IVD) degeneration is a common CFJ OA comorbidity. Non-steroidal anti-inflammatory drug (NSAID) administration is standard for treatment of pain from degenerative joint disease. Potential side effects and tolerance from prolonged administration drive efforts to identify compounds that may be alternatives to or combined with NSAIDs. Agmatine, decarboxylated arginine, reportedly alleviates neuropathic pain, a likely component of OA pain. The objective of this study was to compare treatment response to agmatine and carprofen in dogs with varying degrees of CFJ OA with or without IVD degeneration and to test the hypothesis that agmatine improves hindlimb use comparably to carprofen and more than placebo.

**Methods:** Nine hound-type dogs received oral carprofen (4.4 mg/kg, sid) for 7 days. Six months later, oral agmatine sulfate (25 mg/kg, bid) or placebo (hydroxypropyl methylcellulose, bid) was administered to the same dogs for 28 days with a 2 week washout period between treatments. Validated pain assessment scores were measured before treatment and every seven days throughout the treatment periods. Serum chemistry levels and ground reaction forces (GRF) were quantified before and after each treatment period. A board-certified radiologist quantified radiographic CFJ OA based on Orthopedic Foundation for Animals criteria and IVD degeneration on magnetic resonance images. GRFs were compared among treatments at each time point and among time points for each treatment.

**Results:** There were no detectable adverse effects with any treatment. Significant results included improved GRFs in dogs with mild CFJ OA (*N* = 3) following agmatine administration compared to carprofen or placebo and a trend for improved GRFs in dogs with moderate CFJ OA (*N* = 2) following carprofen vs. agmatine or placebo. Neither agmatine nor carprofen improved GRFs in dogs with severe CFJ OA (*N* = 4). The GRFs improved in dogs with IVD degeneration (*N* = 3) following carprofen treatment compared to agmatine or placebo regardless of CFJ OA score, but no effect was observed in dogs with normal lumbar spines (*N* = 6).

**Conclusions:** Results support agmatine over carprofen treatment to improve limb use in dogs with early or mild CFJ OA, while carprofen may be the better choice for dogs with moderate CFJ OA or IVD degeneration regardless of CFJ OA severity.

## Introduction

Osteoarthritis (OA) affects up to 12 million dogs in the U.S., the majority of which are older than 8 years (1, 2). Canine hip dysplasia (CHD) is among the most common degenerative orthopedic conditions in canine companions and joint changes are biphasic, starting with coxofemoral joint (CFJ) capsule, periarticular and articular damage, and followed by joint degeneration and OA (1, 3–6). Dogs with CFJ laxity associated with CHD are reported to have a higher risk of OA in other joints including vertebral (7–9). Intervertebral disc (IVD) degeneration is a frequent comorbidity or component of CHD pathology (8). Similar to CFJ changes, IVD degeneration progresses with advancing age with many dogs over 7 years old affected by nucleus pulposus degeneration and/or IVD protrusion (10, 11). Pain from IVD degeneration can be intrinsic to the disc or extrinsic radicular or facet joint pain (12, 13).

Customized pain management with non-steroidal anti-inflammatory drugs (NSAIDs) is a standard part of conservative or post-surgical treatment regimens for dogs with CHD associated joint changes and/or IVD degeneration (14, 15). Although life-threatening adverse effects are rare, a recent assessment of causality showed that NSAIDs have the potential to cause or exacerbate digestive tract, renal, and hepatobiliary disorders (16). Of NSAIDs approved for use in dogs, carprofen is one of the mainstays. In a recent study, signs of pain were lower in 74% of canine OA patients after 120 days of carprofen while 5% had adverse gastrointestinal effects associated with the drug (17). With a 100-fold higher selectivity for cyclooxygenase 2 vs. 1, adverse effects are less prevalent than with other less specific NSAIDs (18).

Contributions to joint pain are known to be both neurogenic and musculoskeletal in origin (19). Current knowledge supports that contributions of each wax and wane throughout the course of the disease, though neuropathic pain may predominate during the early stages based on information from human OA (20). Pain from OA is multifaceted and thought to be exacerbated by central sensitization that facilitates nociceptive transmission and increased gain in the spinal cord (21). In human patients, mechanical joint hyperesthesia may be a consequence of altered pain modulation at the spinal or supraspinal level (19). The phenomenon was recently confirmed in dogs undergoing total hip replacement for painful CFJ disease (21). Additionally, it is proposed that neuropathic pain is the reason some human patients with minor CFJ changes do not respond to conventional analgesic treatment (20). Comprehensive therapy to specifically target distinct sources of pain will improve the mobility of dogs with CFJ OA with or without IVD degeneration.

Agmatine is a ubiquitous compound formed during the natural process of arginine decarboxylation (22, 23). The compound is proposed to have many roles in physiologic homeostasis including neuromodulation, neuroprotection, mitochondrial function, and antioxidant and antineoplastic effects (24–26). Agmatine administration alleviates thermal allodynia and hyperalgesia in rat models of both constrictive nerve injury and streptozocin (STZ) induced diabetes (27, 28), and the effects on neuropathic pain are thought to be dose-dependent (28, 29). Proposed mechanisms of action include stimulation of α_2_-adrenergic and imidazoline receptors combined with inhibition of N-methyl-D-aspartate receptors and all isoforms of nitric oxide synthase (30–33). Given the potential for alleviation of neuropathic joint pain, agmatine may be a useful adjunct to current mechanisms for improving hind limb use in dogs with CFJ OA with or without IVD degeneration.

Based on the current understanding of the complexity of CFJ pain from OA and the potential for pain emanating from IVD degeneration, we hypothesized that agmatine improves hind limb use comparably to carprofen and more than placebo. To test this hypothesis, a prospective, randomized, crossover study was performed to quantify changes in validated subjective pain scores, serum chemistry values and hind limb ground reaction forces (GRFs) following oral administration of carprofen, agmatine, and placebo to dogs with varying degrees of CFJ OA with or without IVD degeneration.

## Materials and Methods

### Animals

Nine adult, purpose-bred, hound-type dogs from a teaching and research colony owned by Louisiana State University were included in the study (34, 35). Dogs were housed in temperature-regulated, 1.2 × 2.4 m runs, with daily 1 h free play sessions for the duration of the study. They had free-choice kibble (Laboratory Canine Diet 5006, LabDiet, St. Louis, USA) and *ad libitum* water. The study was performed in accordance with Institutional and National Institutes of Health regulations governing the treatment of vertebrate animals and initiated after approval by the Institutional Animal Care and Use Committee.

### Study Design

The study was a prospective, randomized, placebo-controlled, crossover design. All dogs were treated with carprofen (4.4 mg/kg, PO, sid; quellin®, BAYER, Leverkusen, Germany) for 7 days. Approximately 6 months later, the same dogs were randomly assigned using a randomized block design to receive agmatine sulfate (25 mg/kg, PO, bid; G-Agmatine®, Gilad & Gilad LLC, Henderson, USA) or hydroxypropyl methylcellulose (PO, bid) for 28 days with a 14 day washout period between treatments. All medication containers were coded, and investigators were unaware of treatment identities until all data collection, reduction and analysis was complete. Prior to and at the end of each treatment phase, blood was drawn by venipuncture to measure plasma levels of aspartate aminotransferase (AST), alanine aminotransferase (ALT), alkaline phosphatase (ALP), blood urea nitrogen (BUN), and creatinine (CREAT), and ground reaction forces (GRFs) were quantified. Radiographs were performed prior to study initiation and magnetic resonance imaging (MRI) after the study conclusion. As part of the monitoring regimen for adverse drug reactions, all dogs were assessed prior to and after every 7 days of treatment using the Colorado State University Canine Acute Pain Scale during carprofen administration and with the Canine Brief Pain Inventory and the Glasgow Composite Pain Scale Short Form during agmatine and placebo administration (36–38). The same investigators performed all assessments, and they were not aware of specific treatment administration at any point during the study.

### Radiographs

Ventrodorsal extended hip pelvic radiographs were performed with the dogs under general anesthesia. All dogs were fasted for at least 8 h prior to anesthesia. They were premedicated with butorphanol (0.3 mg/kg, IM) and dexmedetomidine (3 μg/kg, IM). Propofol (3.5 mg/kg, IV) was administered for induction and tracheal intubation. Anesthesia was maintained with isoflurane in oxygen in a semi-closed circle system.

Radiographic changes were quantified by a board-certified radiologist based on Orthopedic Foundation for Animals (OFA) criteria. Numeric values were assigned to each CFJ as follows: 0 (none) = no acetabular dysplasia, no OA; 1 (mild) = acetabular dysplasia, no OA; 2 (moderate) = acetabular dysplasia, OA; or 3 (severe) = complete femoral head luxation, marked OA (39). Both joints in each dog were assigned a value and the highest value of the two was used as the OA severity score. This was to ensure accurate representation of OA severity for the duration of the study.

### Magnetic Resonance Imaging

Magnetic resonance imaging scans were performed on all dogs at the end of the study. Imaging was performed with the dogs positioned in dorsal recumbency under general anesthesia identically to radiographic imaging. Sagittal and transverse T2-W, sagittal fast inversion recovery (FIR), transverse proton density (PD), and dorsal T1-W sequences (Hitachi Echelon 1.5 T, Twinsburg, OH, USA) were performed. Images were evaluated by the same board-certified radiologist as above for presence or absence of disc dehydration, disc bulging, and spinal cord compression in the lumbar spine. The presence of any sign was considered confirmation of IVD degeneration.

### Gait Kinetics

Kinetic gait analysis was performed prior to and after each treatment regimen. The process was similar to that described previously (40). Briefly, a 900 × 900 mm force platform (Model # OR6-WP-1000, Advanced Mechanical Technology, Inc., Watertown, USA) embedded in the center of a runway was used for all trials. A series of five retroreflective photocell sensors (Mek92–PAD, Joslyn Clark Controls, Inc., Lancaster, USA) were used to calculate trial velocity and acceleration. The force platform surface has the same color and texture as the runway. Dogs were conditioned to the force platform and experienced handlers trotted them for all trials. A trial was considered successful if a fore foot contacted the force platform followed by contact of the ipsilateral hind foot at a velocity of 1.70–2.40 m/s and acceleration of 0.9 to −0.9 m/s^2^, a comfortable trotting pace for the study subjects. Three trials that varied <5% in velocity were selected for each dog at each time point. All trials were recorded at a rate of 1,000 Hz and processed with commercially available software (Acquire v7.3, Sharon Software Inc., Dewitt, USA). Measured forces included y (craniocaudal, braking and propulsion) and z (vertical) peak force and impulse. The mean of the GRF values for both right and left limbs was used as a single value for individual dogs (34). Percent change in each GRF measure was calculated as [(GRF after treatment–GRF at baseline)/(GRF at baseline)] × 100.

### Statistical Analysis

Multivariate analysis of variance models was used to compare fixed effects of treatment with random effects of dog and time. Significance level was set at *p* < 0.05. Data was presented as mean ± SEM.

## Results

### Animals

Three males and 6 females, 3.7 ± 0.6 years of age (range 2–6 years) with a body weight of 26.6 ± 0.9 kg (range 23–30.4 kg), were included in the study. All dogs had evidence of CFJ pathology (Table 1). Three dogs had abnormal lumbar spine MRI findings. Serum chemistry results were within normal limits and there was no impact on subjective pain evaluations with any treatment. Subjectively, none of the dogs favored any limbs or showed detectable signs of distress or acute pain over the course of the study.

**Table 1:** Study dog information including CFJ OA grade and MRI findings.

**Age (yrs)**	**Sex**	**CFJ OA**	**Abnormal findings**	
			**Dehydrated disc**	**Disc bulging**
6	M	3	none	no
5	F	3	L1-2, LS	minimal
5	M	3	none	no
2	F	3	none	no
5	F	2	LS	no
2	F	2	none	no
4	F	1	LS	yes
2	M	1	none	no
2	F	1	none	no

*L, lumbar; LS, lumbosacral*.

### Gait Kinetics

**Figure 1:**
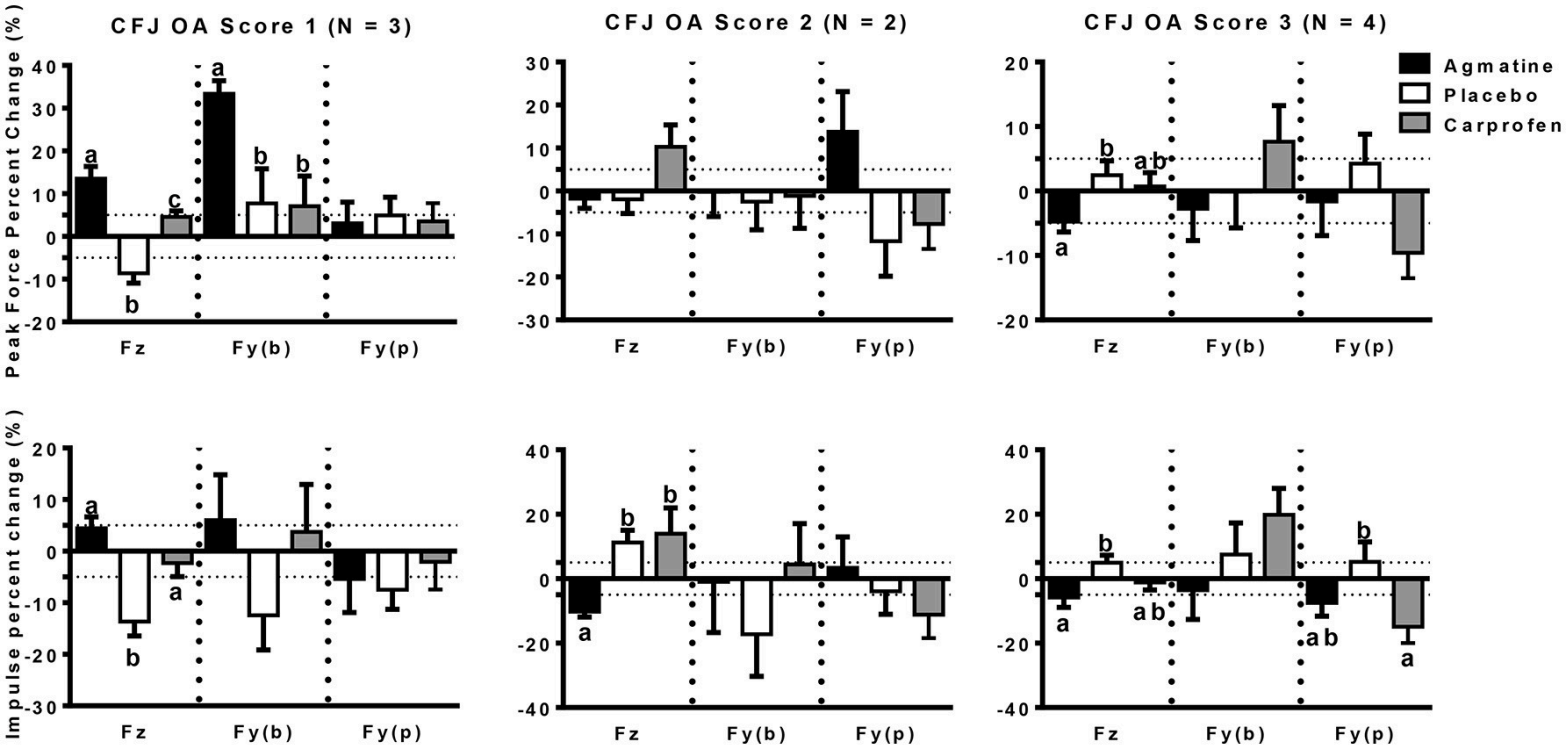
Peak force (PF: upper panels) and impulse (Imp: lower panels) percent change (mean +/- SEM) in dogs with different radiographic coxofemoral joint osteoarthritis scores (CFJ OA score 1: left panels; score 2: central panels; score 3: right panels) following treatment with agmatine (black bars), placebo (white bars), or carprofen (gray bars). In each panel, Fz represents vertical force (left 3 bars), Fy(b) represents braking force (central 3 bars), and Fy(p) represents propulsion force (right 3 bars). Columns with different letters are significantly different among treatment groups for the indicated ground reaction force (*p* < 0.05). The dashed horizontal lines demarcate 5% positive (upper) or negative (lower) changes in each ground reaction force.

**Figure 2:**
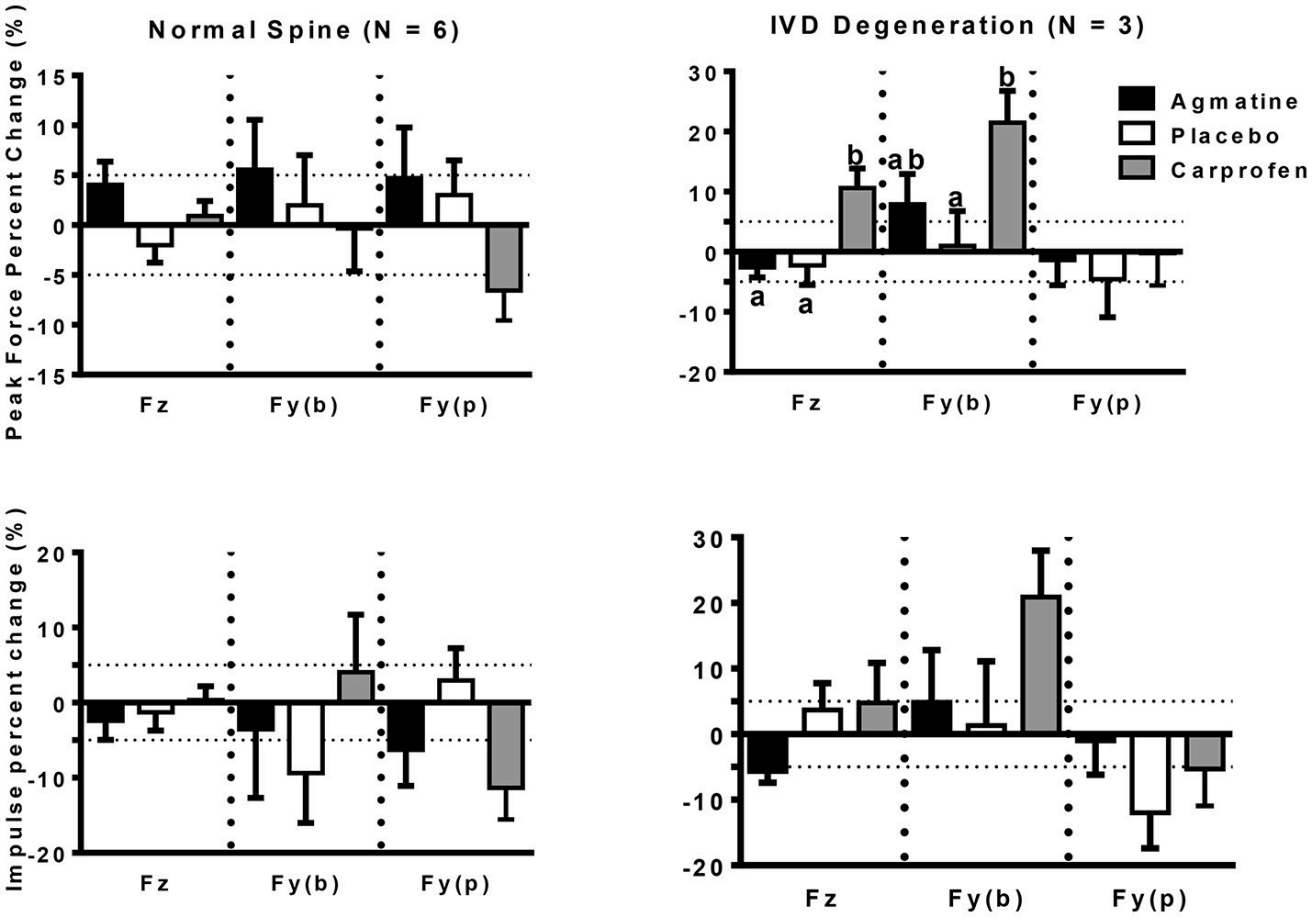
Peak force (PF: upper panels) and impulse (Imp: lower panels) percent change (mean +/– SEM) in dogs with normal spine (left panels) or intervertebral disc degeneration (IVD degeneration: right panels) following treatment with agmatine (black bars), placebo (white bars), or carprofen (gray bars). In each panel, Fz represents vertical force (left 3 bars), Fy(b) represents braking force (central 3 bars), and Fy(p) represents propulsion force (right 3 bars). Columns with different letters are significantly different among treatment groups for the indicated ground reaction force (*p* < 0.05). The dashed horizontal lines demarcate 5% positive (upper) or negative (lower) changes in each ground reaction force.

The primary outcome measures in the investigation were from kinetic gait analysis, the established gold standard for quantification of limb pain (41). Baseline kinetic values were not significantly different between treatments for any of the dogs. Significant findings included higher increases in vertical and braking peak force following treatment with agmatine vs. carprofen or placebo and a greater increase in vertical peak force following treatment with carprofen vs. placebo in dogs with an OA score of 1 (Figure 1, Tables 2, 3). Although change in vertical peak force was greater following treatment with carprofen vs. agmatine or placebo in dogs with an OA score of 2, it was not statistically significant. Notably, vertical peak force increased following carprofen treatment in 1 dog, whereas it did not increase in the other (Supplementary Figure 1). There was little change in GRFs in dogs with an OA score of 3 with any of the treatments; changes were <5% with an exception of a higher change in propulsion impulse after placebo vs. carprofen treatment. In comparisons among dogs with IVD degeneration when considering all OA scores together, vertical peak force increased more after carprofen vs. agmatine or placebo treatment (Figure 2). Additionally, peak braking force increase was greater after carprofen vs. placebo treatment. There were no differences in GRF percent changes among treatments in dogs with no detectable IVD degeneration.

**Table 2:** Percent change of GRF in peak force (PF) and impulse (Imp) represented as mean ± SEM.

		**Fz - PF**	**Fz - Imp**	**Fy(b) - PF**	**Fy(b) - Imp**	**Fy(p) - PF**	**Fy(p) - Imp**
CFJ OA 1	A	13.55 ± 2.84	4.46 ± 2.17	33.36 ± 3.08	6.02 ± 8.79	3.06 ± 4.99	−5.41 ± 6.48
	P	−8.63 ± 2.32	−13.65 ± 2.78	7.74 ± 8.07	−12.44 ± 6.71	4.92 ± 4.24	−7.49 ± 3.79
	C	4.59 ± 1.40	−2.31 ± 2.65	7.08 ± 7.05	3.72 ± 9.22	3.51 ± 4.28	−2.05 ± 5.40
CFJ OA 2	A	−1.80 ± 2.21	−10.21 ± 1.70	−0.19 ± 5.75	−0.95 ± 15.7	13.78 ± 9.35	3.34 ± 9.60
	P	−1.92 ± 3.30	11.25 ± 3.79	−2.48 ± 6.55	−17.26 ± 13.05	−11.65 ± 8.18	−3.90 ± 7.14
	C	10.29 ± 5.06	13.93 ± 8.04	−1.13 ± 7.52	4.46 ± 12.66	−7.67 ± 5.83	−11.16 ± 7.30
CFJ OA 2 Dog 1	A	−6.62 ± 2.18	−10.19 ± 3.17	−1.72 ± 6.12	−28.03 ± 9.77	13.70 ± 8.47	16.13 ± 9.68
	P	−6.10 ± 4.93	22.73 ± 2.25	2.26 ± 8.24	20.63 ± 10.41	−28.36 ± 4.25	−21.58 ± 4.05
	C	21.48 ± 7.84	38.89 ± 4.20	11.33 ± 5.42	19.20 ± 13.55	−5.65 ± 11.41	−0.40 ± 10.92
CFJ OA 2 Dog 2	A	3.03 ± 2.72	−10.23 ± 1.66	1.35 ± 10.32	26.14 ± 23.86	13.90 ± 25.67	−3.05 ± 13.34
	P	2.25 ± 4.07	−0.24 ± 2.30	−7.22 ± 10.57	−40.00 ± 10.00	13.41 ± 10.39	17.32 ± 7.03
	C	−0.90 ± 1.06	−11.01 ± 4.23	−19.83 ± 12.53	−25.00 ± 0.00	−9.69 ± 4.22	−21.93 ± 8.29
CFJ OA 3	A	−4.76 ± 1.58	−5.83 ± 3.07	−2.75 ± 4.95	−3.54 ± 9.11	−1.62 ± 5.31	−7.51 ± 4.11
	P	2.44 ± 2.25	4.94 ± 2.38	−0.08 ± 5.63	7.47 ± 9.81	4.24 ± 4.56	5.27 ± 6.17
	C	6.49 ± 2.53	−0.79 ± 2.75	14.92 ± 7.97	19.76 ± 9.82	−12.41 ± 5.18	−19.60 ± 6.87
IVD degeneration	A	−2.61 ± 1.68	−5.69 ± 1.70	7.89 ± 5.03	4.86 ± 7.95	−1.40 ± 4.18	−1.01 ± 5.16
	P	−2.26 ± 3.25	3.70 ± 4.08	0.98 ± 5.78	1.32 ± 9.80	−4.58 ± 6.32	−11.98 ± 5.43
	C	10.59 ± 3.27	4.75 ± 6.11	21.49 ± 5.28	20.91 ± 7.08	−0.24 ± 5.36	−5.30 ± 5.65
Normal spine	A	4.04 ± 2.31	−2.40 ± 2.53	5.57 ± 5.01	−3.54 ± 9.14	4.71 ± 5.07	−6.29 ± 4.79
	P	−2.01 ± 1.76	−1.29 ± 2.42	2.00 ± 5.02	−9.38 ± 6.65	3.01 ± 3.47	2.94 ± 4.29
	C	5.49 ± 1.91	5.37 ± 1.29	3.41 ± 5.52	2.40 ± 9.48	−5.74 ± 4.49	−9.16 ± 6.09

*GRF from Agmatine (A), placebo (P), and Carprofen (C) treatment groups were compared based on different grades of radiographic CFJ OA (1–3) and presence or absence of IVD degeneration (IVD Degeneration or Normal Spine)*.

*Fz: vertical force, Fy(b/p): craniocaudal force (braking/propulsion)*.

**Table 3:** P-values of multiple comparison for GRF in peak force (PF) and impulse (Imp) between Agmatine (A), placebo (P), and Carprofen (C) treatment groups based on different grades of radiographic CFJ OA (1–3) and presence or absence of IVD degeneration (IVD Degeneration or Normal Spine).

		**Fz - PF**	**Fz - Imp**	**Fy(b) - PF**	**Fy(b) - Imp**	**Fy(p) - PF**	**Fy(p) - Imp**
CFJ OA 1	A vs. C	**0.0148**	0.0507	**0.0171**	0.8726	0.9974	0.9484
	A vs. P	**<0.0001**	**<0.0001**	**0.0153**	0.398	0.9572	0.8911
	C vs. P	**0.0003**	**0.0016**	0.9889	0.4812	0.9715	0.7093
CFJ OA 2	A vs. C	0.0747	**0.0002**	0.9847	0.9434	0.1634	n/a
	A vs. P	0.9997	**0.001**	0.9636	0.6641	0.1085	0.9556
	C vs. P	0.071	0.8707	0.9966	0.5842	0.9396	0.9558
CFJ OA 2 Dog 1	A vs. C	**0.0067**	**<0.0001**	0.3785	**0.0413**	0.3925	0.4813
	A vs. P	0.9974	**<0.0001**	0.9085	0.0505	**0.0303**	**0.0461**
	C vs. P	**0.0077**	**0.0093**	0.6148	0.9961	0.167	0.1904
CFJ OA 2 Dog 2	A vs. C	0.6114	0.9808	0.426	0.2373	0.3508	0.3996
	A vs. P	0.9799	0.0719	0.8316	**0.0426**	0.4288	0.3802
	C vs. P	0.7268	0.0507	0.7288	0.8545	0.0654	**0.0472**
CFJ OA 3	A vs. C	0.1244	0.4206	0.2637	0.2182	0.6064	0.5357
	A vs. P	**0.0305**	**0.013**	0.9835	0.6978	0.5832	0.2109
	C vs. P	0.8116	0.2289	0.4124	0.6685	0.0915	**0.0186**
IVD degeneration	A vs. C	**0.0057**	0.076	**0.0471**	0.3685	0.955	0.876
	A vs. P	0.9959	0.1212	0.6198	0.9309	0.9568	0.4192
	C vs. P	**0.0072**	0.9729	**0.0066**	0.3171	0.8214	0.6731
Normal spine	A vs. C	0.4402	0.6728	0.3058	0.7928	0.1084	0.3971
	A vs. P	0.0564	0.9347	0.5778	0.8901	0.9348	0.5659
	C vs. P	0.5053	0.8726	0.8927	0.412	0.1384	0.0572

*Fz: vertical force, Fy(b/p): craniocaudal force (braking/propulsion). Significant values were represented in bold letter. n/a indicates not available*.

## Discussion

The results of this study suggest that agmatine may improve hind limb use in dogs with early CFJ OA while carprofen may be more effective in dogs with moderate joint degeneration or IVD degeneration regardless of CFJ OA. Based on these results, the hypothesis is rejected, and it appears that agmatine may be warranted for dogs with CFJ pain from early joint changes, but carprofen is the therapeutic choice for those with more advanced disease with or without concurrent IVD pathology.

The goal of this study was to evaluate gait after a meaningful period of therapeutic drug levels. Based on existing information, carprofen reaches maximum plasma levels in dogs after 1 h (42). As indicated previously, investigators were not aware of the identity of the medications tested in the study prior to completion of data collection, reduction, and analysis. This is in spite of the fact that carprofen was administered for a shorter period of time given concerns about potential for development of gastrointestinal problems in the specific dog population included in the study. Signs of gastrointestinal adverse effects can occur within 2–4 weeks of carprofen administration (43), and the American Animal Hospital Association recommends inclusion of a minimum 7 day washout period between NSAIDs if continual treatment is required (44). The pharmokinetics of agmatine in dogs have yet to be established, so the duration and dose were derived from existing information in other species (23, 45), some of which include direct intrathecal or intraperitoneal administration (27, 28). Neuropathic pain without OA in humans was alleviated following 14 days of agmatine (23). The adminstration period in this study was doubled to help ensure sufficient time with therapeutic agmatine levels for chronic musculoskeletal pain. Based on study findings, agmatine did not have any adverse effects over the duration of the study which appeared to be sufficient to reduce pain from mild CFJ OA.

The fact that the time periods of drug administration were not identical is a limitation of the study. It is possible that gait parameters in dogs with mild CFJ OA might have improved with longer carprofen treatment based on the tendency (*p* = 0.11) for increased peak vertical force measured in dogs with coxofemoral and elbow joint OA after 28 days of half the dose (2 mg/kg) used in this study (46). Additionally, oral administration of carprofen reportedly improved clinical signs in dogs with OA in various joints after 4 months of administration (17). Clear improvement in one dog with moderate OA and IVD degeneration following carprofen treatment while another with moderate OA and no IVD degeneration did not respond suggests that the duration of carprofen administration was sufficient for pain relief. However, the small sample size and lack of serum agmatine levels precludes any conclusions on this point.

Quantification of GRFs is an established mechanism to assess therapeutic efficacy for limb and back pain in dogs and other species (34, 47, 48). Careful quality control is necessary to ensure meaningful results and this was implicit in study standards that required variation of 5% or less among trials. The crossover nature of the study provided a direct comparison of treatments for each dog. The MRI assessments performed after the last study phase ensured that the IVD degeneration scores reflected the current condition of the lumbar spine. While it is possible that OA and/or IVD progressed slightly over the study period, gait kinetic values, the established gold standard for evaluation of joint pain, were the primary outcome measures in this study (41). The fact that baseline kinetic parameters did not change significantly between treatments makes it unlikely that CFJ OA or IVD degeneration changed appreciably. The small number of dogs with evidence of IVD degeneration or moderate OA allows only a presumption of superior carprofen pain control for the conditions. Additional studies with a much larger population containing various combinations of spine and CFJ pathology are necessary to confirm the benefits of carprofen over agamatine for IVD degeneration and moderate CFJ OA.

Agmatine may provide a mechanism to reduce CFJ pain during initial disease stages and carprofen for later stages, or combined therapy may be more effective than either alone. A multi-modal approach to pain management offers a wider range of options to maintain canine activity levels as needs change (49, 50). Additional work to determine the effects of combined therapies may show synergistic effects based on existing work that supports administration of agmatine with other compounds that inhibit N-methyl-D-aspartate receptors or nitric oxide synthase (45). Combining agmatine with gabapentin, reported to be beneficial for canine neuropathic pain, may be another option (51, 52).

## Conclusions

This pilot study establishes the potential benefits of agmatine treatment for early stage canine CFJ OA. Agmatine administration may conceivably improve limb use and associated quality of life. Further investigation is required to confirm the promising findings of this early pilot study in a heterogeneous canine population. Future work may show benefits of treatment regimens that combine agmatine with established pain management, including NSAIDs. This study establishes one potential dosage, treatment route and interval for effective pain relief in dogs with early stage CFJ OA. The information is a valid starting point to test the compound for management of discomfort for distinct conditions in diverse canine companions.

## Data Availability Statement

The datasets supporting the conclusions of this article are included within the article and its additional files. All datasets used and/or analyzed during the current study are available from the corresponding author on reasonable request.

## Author Contributions

TT, RK, and ML contributed to the study design, data collection, data reduction, analysis and interpretation, and manuscript preparation. CT and NR contributed to data collection and interpretation and manuscript preparation. GG and RA contributed to manuscript editting. All authors read and approved the final version of the manuscript.

### Conflict of Interest Statement

GG is co-owner and CEO of Gilad & Gilad, LLC the company that provided the agmatine treatment used in this study (G-Agmatine R, US Patent No. 8916612). The remaining authors declare that the research was conducted in the absence of any commercial or financial relationships that could be construed as a potential conflict of interest.

## Supplementary Material

Supplementary Figure 1 Peak force (PF: upper panels) and impulse (Imp: lower panels) percent change (mean +/- SEM) in 2 dogs (dog 1 and 2) with coxofemoral joint osteoarthritis scores 2 (CFJ OA score 2) following treatment with agmatine (black bars), placebo (white bars), or carprofen (gray bars). In each panel, Fz represents vertical force (left 3 bars), Fy(b) represents braking force (central 3 bars), and Fy(p) represents propulsion force (right 3 bars). Columns with different letters are significantly different among treatment groups for the indicated ground reaction force (*p* < 0.05). The dashed horizontal lines demarcate 5% positive (upper) or negative (lower) changes in each ground reaction force.

## Abbreviations

CFJ: coxofemoral joint
OA: osteoarthritis
CHD: canine hip dysplasia
IVD: intervertebral disc
NSAID: non-steroidal anti-inflammatory drug
GRF: ground reaction force
AST: aspartate aminotransferase
ALT: alanine aminotransferase
ALP: alkaline phosphatase
BUN: blood urea nitrogen
CREAT: creatinine
MRI: magnetic resonance imaging
OFA: Orthopedic Foundation for Animals
FIR: fast inversion recovery
PD: proton density.
