# Supplementary figures and images for: Agmatine for Pain Management in Dogs With Coxofemoral Joint Osteoarthritis: A Pilot Study
Source: Front Vet Sci. 2018 Dec 12;5:311. doi: 10.3389/fvets.2018.00311 (PMC6315183; doi:10.3389/fvets.2018.00311)

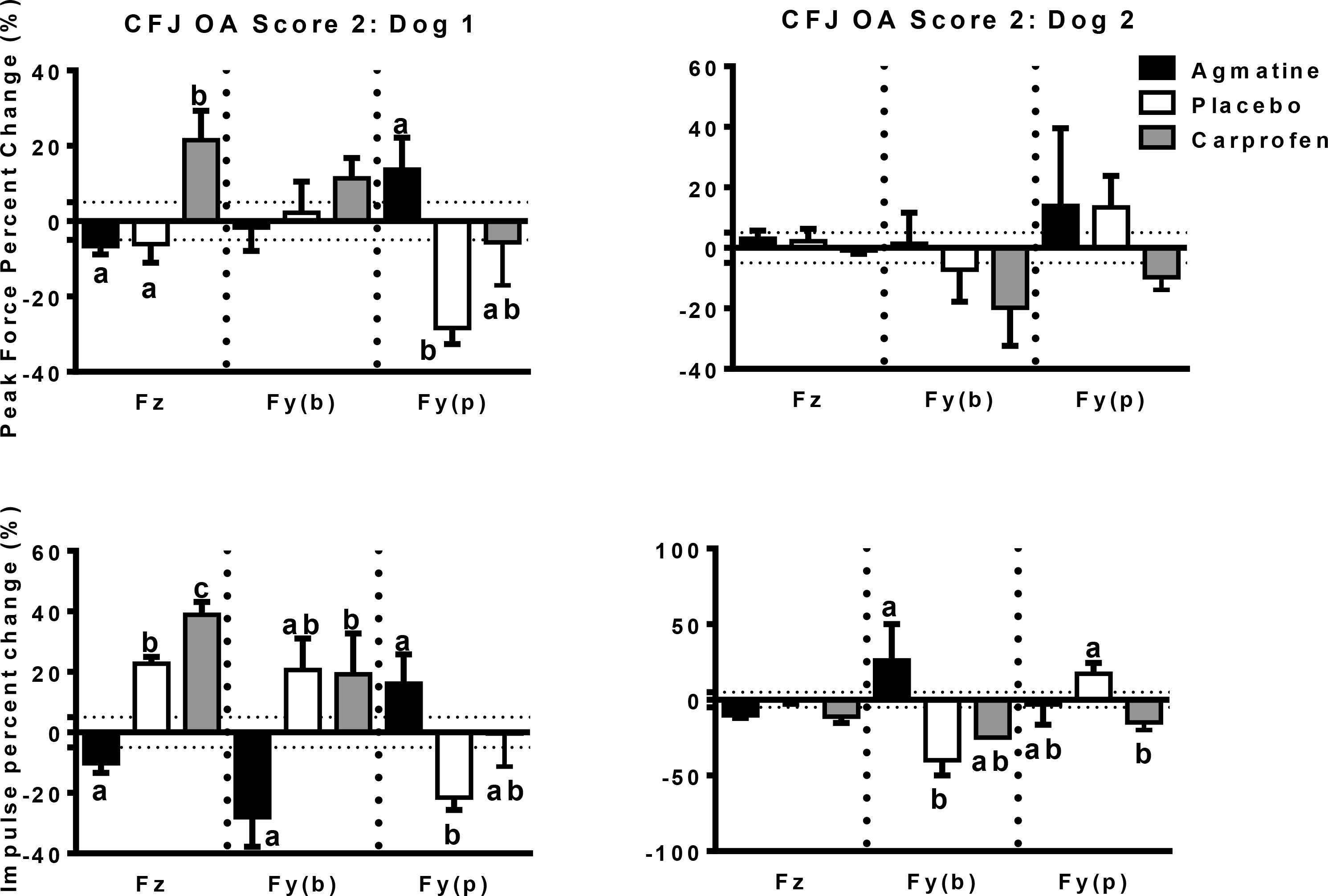

Supplement: Supplementary Figure 1 — Peak force (PF: upper panels) and impulse (Imp: lower panels) percent change (mean +/- SEM) in 2 dogs (dog 1 and 2) with coxofemoral joint osteoarthritis scores 2 (CFJ OA score 2) following treatment with agmatine (black bars), placebo (white bars), or carprofen (gray bars). In each panel, Fz represents vertical force (left 3 bars), Fy(b) represents braking force (central 3 bars), and Fy(p) represents propulsion force (right 3 bars). Columns with different letters are significantly different among treatment groups for the indicated ground reaction force (p < 0.05). The dashed horizontal lines demarcate 5% positive (upper) or negative (lower) changes in each ground reaction force. [file Image_1.tif]
